# Cytopathological analysis of bronchoalveolar lavage fluid in patients with and without HIV infection

**DOI:** 10.1186/s12890-022-01851-0

**Published:** 2022-02-08

**Authors:** Xiang-mei Chen, Lei Sun, Kun Yang, Jia-min Chen, Liang Zhang, Xiao-yi Han, Xingang Zhou, Zhi-yuan Ma, Man Li, Hong-xin Zhao, Li-ming Qi, Peng Wang

**Affiliations:** 1 Department of Pathology, Beijing Ditan Hospital, Capital Medical University, No. 8 Jing Shun East Street, Chaoyang District, Beijing, 100015 People’s Republic of China; 2 Center for Infectious Diseases, Beijing Ditan Hospital, Captial Medical University, Beijing, 100015 People’s Republic of China

**Keywords:** HIV/AIDS-related lung disease, Bronchoalveolar lavage fluid, Cytopathology, Pathogen morphology

## Abstract

**Background:**

Human immunodeficiency virus/acquired immunodeficiency syndrome (HIV/AIDS) infection can lead to a broad spectrum of lung diseases, including infectious diseases and tumors. Recently, with the wide application of bronchoscopes and cytopathology of bronchoalveolar lavage fluid (BALF), the diagnostic efficiency of lung diseases has improved. The present study focuses on analyzing the cytopathologic characteristics of BALF in the diagnosis of HIV/AIDS-related lung disease and comparing the lung disease spectrum between HIV and HIV-uninfected patients.

**Methods:**

BALF specimens were collected from 2211 patients. Using ThinPrep liquid-based technology, the cytologic smears were prepared by staining with Hematoxylin and Eosin (HE), Gomori's methenamine silver (GMS), and Periodic Acid Schiff (PAS), acid-fast and immunocytochemical (ICC) staining. Real-time PCR was used to detect cytomegalovirus (CMV) and *Mycobacterium tuberculosis (M. tuberculosis)* in the remaining BALF. PCR-reverse dot hybridization was used for mycobacterial species identification.

**Results:**

From the 2211 BALF specimens, 1768 (79.96%) were specimens from HIV-infected patients, and 443 (20.04%) were speciments from HIV-uninfected patients. The HIV-infected patients with a median age of 38.5 ± 11.3 years were markedly younger than the HIV-uninfected patients (52.9 ± 14.9 years) (*p* < 0.01). We found that 1635 (92.5%) HIV-infected patients were males, showing a prominently higher proportion than those without HIV infection (71.1%) (*p* < 0.01). Meanwhile, 1045 specific lesions were found in 1768 HIV-infected patients (59.1%), including 1034 cases of infectious diseases and 11 neoplastic lesions, also exhibiting a distinctly higher proportion compared to the HIV-uninfected patients (12.2%) (*p* < 0.001). For the HIV-infected group, a distinctly higher proportion of single infection lesions (724/1768, 41%) was noted than the HIV-uninfected group (14/443, 3.2%) (*p* < 0.001). Among single infection lesions, the most common was Cytomegalovirus(CMV) infection (20.9%) for the HIV-infected group, followed by *Pneumocystis jiroveci*(PJ) (13.0%), Fungal (3.5%), and Mycobacterial infections (3.4%), of which *M. tuberculosis* infection accounted for 3.1%. Double infections (300/1768, 17.0%) and Triple infections (10/1768, 0.6%) were found only among the patients with HIV. The malignancies among HIV-infected patients included adenocarcinomas (0.22%), small cell carcinomas (0.2%), squamous cell carcinomas (0.1%), and diffuse large B-cell lymphoma (0.1%). HIV-infected patients exhibited a significantly lower incidence of neoplastic lesions (0.6% vs. 9.0%) than the HIV-uninfected patients (*p* < 0.001).

**Conclusions:**

There was a significant difference in the spectrum of lung diseases between HIV-infected and non-infected patients diagnosed by BALF cytopathology.

## Background

There are two major categories of HIV/AIDS-related pulmonary diseases: infections and tumors. Respiratory symptoms such as cough, chest tightness, shortness of breath, and imaging lung shadow are common clinical manifestations, especially in HIV infection. Recently, bronchoalveolar lavage fluid (BALF) cytopathological examinations based on fiberoptic bronchoscopy have been increasingly used in clinical applications. It is possible to instantly assess BALF smears and repeat the assessment procedure a few times so that further materials for immunocytochemistry and special staining can be obtained. Besides, the component residues of cytological smears can be used for PCR or Metagenomic studies.

For patients with HIV infection, it is crucial to examine and diagnose BALF cytologically. Thus, the present study attempts to clarify the utility of BALF in assessing the characteristics and clinical diagnostic value in HIV/AIDS-related lung diseases.

## Methods

2211 BALF specimens were collected from HIV-infected and non-infected patients treated at the Pathology Department of Beijing Ditan Hospital, Capital Medical University between October 2008 and October 2020. The detailed demographic information of patients from both the pathological reports and the clinician-submitted laboratory requisitions were obtained. Our research protocol was approved by the Ethics Committee of Beijing Ditan Hospital, Capital Medical University in China(2021–022-01). Informed consent was obtained from all individual participants included in the study. Written informed consent was obtained from the participants by physicians.

The patients were routinely prepared, 2% lidocaine (15–20 mL) was used for anesthesia by nebulization, and the examination was performed using the BF-260 electronic bronchoscope (Olympus, Japan). Bronchoalveolar lavage is used 100–150 ml of sterile normal saline lavage at 37℃ in which images suggest the presence of infected lobe segments. If the chest X-ray showed pulmonary interstitial changes or did not indicate evident local lobe involvement, the right middle or left lingular lobe was taken as lavage Location, and the negative recovery pressure was 50–80 mm Hg. BALF was packed in a collection bottle coated with silica gel for inspection. Cell smears were prepared using ThinPrep liquid-based cytology technology. After instant fixation in ethyl alcohol (95%), the smears were stained with hematoxylin and eosin (HE), Gomori's methenamine silver (GMS), periodic acid Schiff (PAS), as well as by immunocytochemical and acid-fast techniques for cytological purposes. Preparation of staining reagents, purchased from Sinopharm Chemical Reagent Co., Ltd. China, was accomplished by the Beijing Ditan Hospital's Pathology Department. The remaining BALF was analyzed using the PCR method for human cytomegalovirus (CMV) (Shanghai Zhijiang Biotechnology Co., Ltd. China) and *Mycobacterium tuberculosis (M. tuberculosis)* (Shanghai Zhijiang Biotechnology Co., Ltd. China). PCR-reverse dot hybridization was used for mycobacterial species identification (Shenzhen Yaneng Biotechnology Co., Ltd. China). The diagnostic criteria for mycobacterial infection was defined as same positive results from at least two separate samples. For individual cases with morphological features of infection, metagenomics was applied to detect pathogens.

SPSS software (Version 20.0, IBM, Chicago, USA) analyzed the collected data. Independent Student's t-test was used to compare the age difference between the HIV-infected and uninfected patients. The χ^2^ test was used for the categorical variable analysis, including gender ratio, proportion of each age group and proportion of various positive diagnoses between HIV infected and uninfected groups.. Statistically, p-value < 0.05 was regarded as significant, while continuous data was expressed as mean ± SD.

## Results

### HIV/AIDS-related lung disease prevalence and distribution

Overall, the study comprised 1768 (1768/2211, 80.0%) patients with HIV and 443 (443/2211, 20.0%) patients without HIV. The HIV-infected group (1635/1768, 92.5%) exhibited a higher proportion of male patients than the HIV-uninfected group (315/443, 71.1%) (*p* < 0.001). The HIV-infected patients were aged from 10 to 96 years, and those aged 18–44 showed the highest proportion (1245/1768, 70.4%). When compared to the HIV non-infected patients, this number was remarkably higher (*p* < 0.001). The patients without HIV infection aged between 14 and 92 years, with those aged 45–59, showed the highest proportion (162/443, 36.6%). Compared to the HIV-infected patients, the proportions among 45–59 age range (163/443, 36.6%), 60–74 age range (129/443, 29.1%), and 75 + age range (26/443, 5.9%) were all remarkably higher (Table 1).

**Table 1:** Comparison of demographic profile between patients with and without HIV infection

	HIV infected patients (cases)	HIV uninfected patients (cases)	Total	*t/x*^2^	*P* value
Sex					
Male	1635 (92.5%)	315 (71.1%)	1950	155.403	< 0.001
Female	133 (7.5%)	128 (28.9%)	261		
Age					
Average/Median	38.5 ± 11.3/36	52.9 ± 14.9/54	41.4 ± 13.4/39	61.853	< 0.001
Range	10–96	14–92			
0–17	6 (0.3%)	3 (0.7%)	9	0.997	0.318
18–44	1245 (70.4%)	123 (27.8%)	1368	73.191	< 0.001
45–59	428 (24.2%)	162 (36.6%)	590	27.665	< 0.001
60–74	86 (4.9%)	129 (29.1%)	215	237.406	< 0.001
≥ 75	3 (0.2%)	26 (5.9%)	29	88.895	< 0.001
Total	1768	443	2211		

1045 specific lesions were found in cytological smears of 1768 BALF specimens of HIV patients (59.1%), including 1034 cases of infectious diseases and 11 of neoplastic lesions, exhibiting a prominently higher proportion compared to the patients without HIV (54/443,12.2%) (*p* < 0.001). For the HIV-infected group, a distinctly higher proportion of single infection lesions (724/1768, 41%) was noted than the HIV-uninfected group (14/443, 3.2%) (*p* < 0.001). Double (300/1768, 17.0%) and Triple infections (10/1768, 0.6%) were found only among the patients with HIV. Among single infection lesions, the most common was CMV infection (20.9%), followed by PJ (13.0%), Fungal (3.5%), and Mycobacterial infections (3.4%). Fungal infections in AIDS patients included 41 cases (2.3%) of Mildew, 13 (0.7%) of *Cryptococcus*, 3 (0.2%) of *Talaromyces marneffei*, 3 (0.2%) of *Histoplasma* and 2 (0.1%) of *Actinomycetes*. Mycobacterial infections were found in 60 HIV patients (3.4%), of which 55 cases (3.1%) were *M. tuberculous*, and 5 (0.3%) were non-tuberculous, including infections with *Mycobacterium avium Complex* (MAC, 2 cases), *Mycobacterium kansasii* (*M. kansasii*, 2 cases) and *Mycobacterium szulgai* (*M. szulgai*, 1 case). Compared to HIV-uninfected patients, HIV-infected patients exhibited a markedly higher proportion of CMV (*p* < 0.001), PJ (*p* < 0.001), and fungal infections (p = 0.009). Among double infections, the most common lesions were PJ combined with CMV infections (221, 12.5%), followed by CMV combined with fungal infections (40, 2.3%), CMV combined with mycobacterial infections (24, 1.4%), CMV combined with other bacterial infections (4, 0.2%) and PJ combined with mycobacterial infections (3, 0.2%). Among triple infection lesions, there were 9 cases (0.5%) of PJ, CMV combined with fungal infections, and 1 case of PJ, CMV combined with mycobacterial infection (0.1%) (Table 2). All cases were confirmed by pathogen culture or follow-up clinical treatment.

**Table 2:** Comparison of cytological diagnosis of bronchoalveolar lavage fluid between patients with and without HIV infection

Diagnosis	HIV infected patients(cases)	HIV uninfected patients(cases)	Total	*x*^2^	*P* value
**Specific lesions**	1045 (59.1%)	54 (12.2%)	1099	311.909	< 0.001
** Single infection**	724 (41.0%)	14 (3.2%)	738	227.493	< 0.001
*Pneumocystis jiroveci* infection (PJ)	229 (13.0%)	0	229	64.009	< 0.001
Cytomegalovirus infection(CMV)	370 (20.9%)	0	370	111.342	< 0.001
Mycobacterial infection	60 (3.4%)	9 (2.0%)	69	2.174	0.140
* M. tuberculosis*	55 (3.1%)	9 (2.0%)	64	1.468	0.226
Nontuberculous mycobacteria	5 (0.3%)	0	5	1.256	0.262
Fungal infection	62 (3.5%)	5 (1.1%)	67	6.818	0.009
Mildew	41 (2.3%)	5 (1.1%)	46	2.464	0.116
* Cryptococcosis*	13 (0.7%)	0	13	3.277	0.070
* Talaromyces marneffei*	3 (0.2%)	0	3	0.753	0.386
* Histoplasma*	3 (0.2%)	0	3	0.753	0.386
Actinomycetes	2 (0.1%)	0	2	0.502	0.479
Bacteria infection	3 (0.2%)	0	3	0.753	0.386
** Double infection**	300 (17.0%)	0	300	86.970	< 0.001
PJ combined with CMV infection	221 (12.5%)	0	221	61.525	< 0.001
PJ combined with mycobacterial infection	3 (0.2%)	0	3	0.753	0.386
PJ combined with Fungal infection	8 (0.5%)	0	8	2.012	0.156
CMV combined with mycobacterial infection	24 (1.4%)	0	24	6.080	0.014
CMV combined with Fungal infection	40 (2.3%)	0	40	10.207	0.001
CMV combined with Bacteria infection	4 (0.2%)	0	4	1.004	0.316
**Triple infection**	10 (0.6%)	0	10	2.517	0.113
PJ, CMV combined with fungal infection	9 (0.5%)	0	9	2.264	0.132
PJ, CMV combined with mycobacterial infection	1 (0.1%)	0	1	0.251	0.617
** Neoplastic lesions**	11 (0.6%)	40 (9.0%)	51	111.109	< 0.001
Squamous cell carcinoma	2 (0.1%)	19 (4.3%)	21	65.659	< 0.001
Adenocarcinoma	4 (0.2%)	14 (3.2%)	18	37.765	< 0.001
Small cell carcinoma	3 (0.2%)	6 (1.4%)	9	12.264	< 0.001
Diffuse large B lymphoma	2 (0.1%)	0	2	0.502	0.479
Squamous papilloma	0	1 (0.2%)	1	3.993	0.046
**Non-specific lesions**	723 (40.9%)	389 (87.8%)	1112	311.909	< 0.001
Total	1768	443	2211		

In addition, 11 cases (0.6%) of neoplastic lesions in HIV patients were found, including pulmonary adenocarcinoma (4 cases), pulmonary small cell carcinoma (3 cases), pulmonary squamous cell carcinoma (2 cases), and diffuse large B-cell lymphoma (2 cases), with significantly lower incidences of neoplastic lesions (0.6% vs. 9.0%, *p* < 0.001), squamous cell carcinoma (0.1% vs. 4.3%), Adenocarcinoma (0.2% vs. 3.2%) and small cell carcinoma (0.2% vs. 1.4%) compared to the HIV-negative patients (*p* < 0.001) (Table 2). These were all confirmed by pathological tissue biopsy.

### Pathogens and cell morphology

#### CMV mainly affects alveolar epithelial cells

**Fig. 1:**
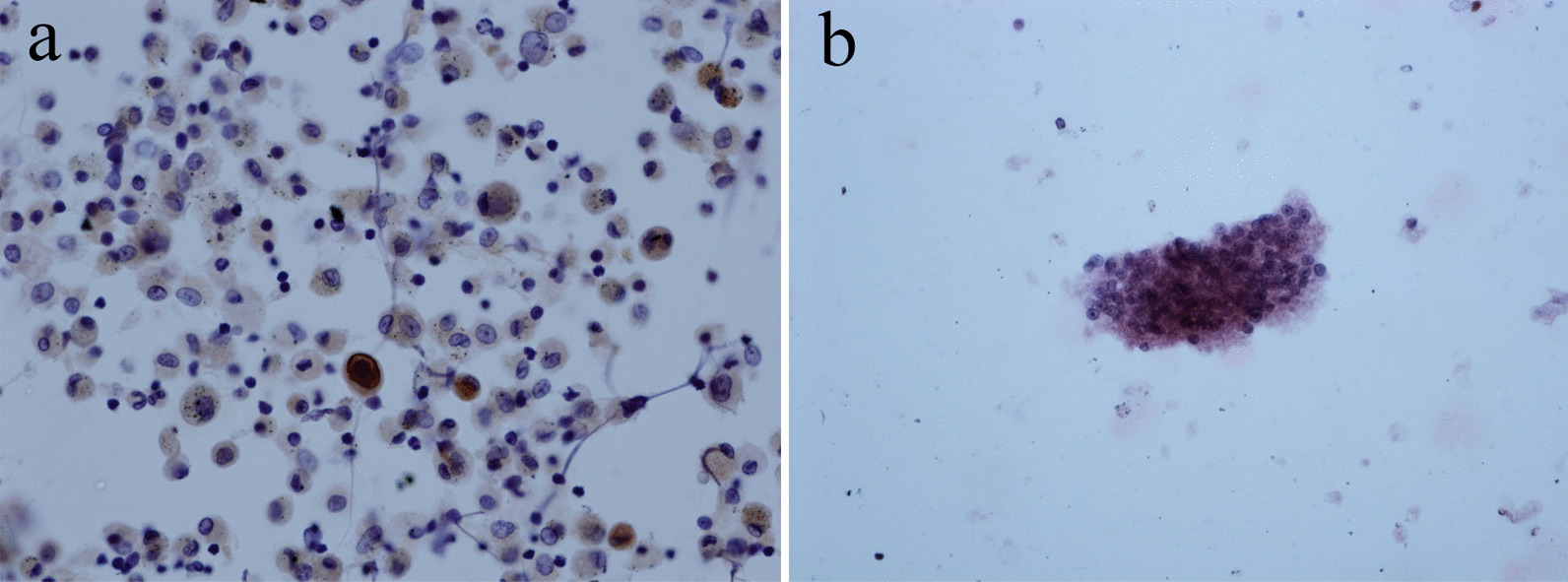
**a** CMV immunocytochemistry show "owl's-eye" like structure. CMV-IHCx40. **b** The walls of the PJ cysts stained with GMS are black, regular or irregular, with obvious nuclei GMSx40

The affected cells increase in volume and form giant cells or nuclear inclusion bodies. These inclusion bodies were purple-blue or purple-red, round or oval, with empty halos around them, showing an "owl's-eye" appearance. Other CMV inclusions were characterized by the affluent rough eosinophilic inclusions of cytoplasm inside the enlarged cells in the absence of clear inclusions of the nucleus. CMV immunocytochemistry showed an "owl's-eye" like structure (Fig. 1a).

#### The main pathological changes of PJ infection were interstitial and alveolar pneumonia

In the HE stained BALF smears, focally distributed pink foam or honeycomb materials were observed. It contained piles of PJ cysts. The walls of the GMS-stained cysts were black, regular, or irregular, with prominent nuclei (Fig. 1b).

#### Mycobacterium BALF smear with HE-staining showed a small number of epithelioid cells and fibrous tissue cells, and a small amount of caseous necrosis was seen in some cases

**Fig. 2:**
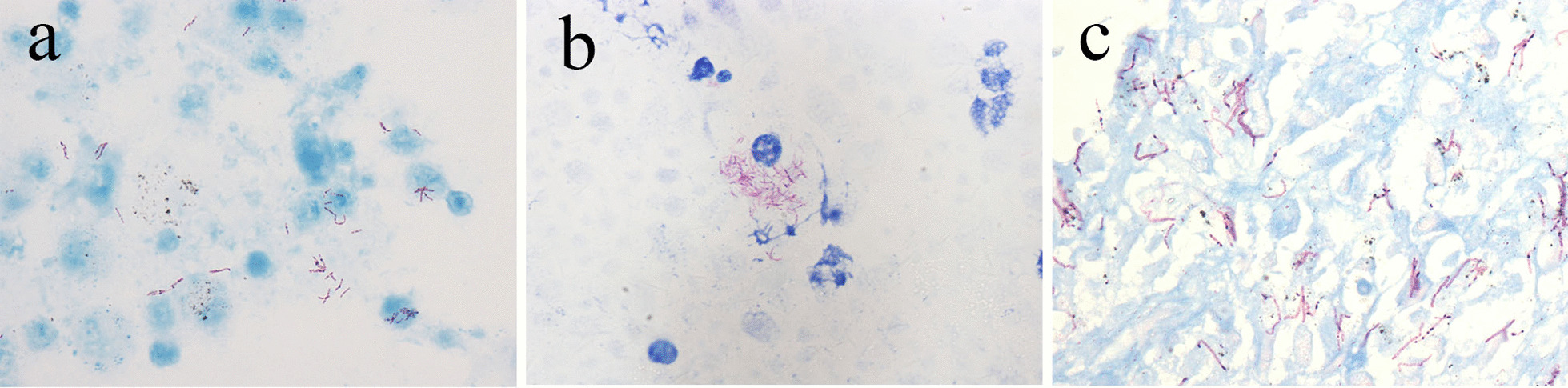
**a** *Mycobacterium tuberculosis* showed red, rod shaped, slender and slightly curved mycobacteria AFCx100. **b** *Mycobacterium avium* Complex were mainly located in the cytoplasm of macrophages. AFCx100. **c** *Mycobacterium kansasii* demonstrated long slender bacilli, often curved or folded and showed irregular coarse beading. AFCx100

In Fig. 2a, red, slightly curved, and slender bacilli-form mycobacteria were revealed upon acid-fast staining of *Mycobacterium tuberculosis*. Smears of Mycobacterium avium Complex (MAC) infections showed macrophages with foamy cytoplasm; MAC was mainly located in the cytoplasm of these macrophages, which could also be shown by acid-fast staining (Fig. 2b). *Mycobacterium kansasii* was confirmed by lung biopsy and a mycobacterial typing test. Histology showed granulomatous inflammation, histiocyte proliferation with lymphocyte infiltration without necrosis. As revealed by acid-fast stain, elongated rod-like bacilli were present, beaded irregularly and coarsely, often folded or curved (Fig. 2c).

#### Fungal infections mainly included Mildew, Cryptococcus, Talaromyces marneffei, Histoplasma, and Actinomycetes

**Fig. 3:**
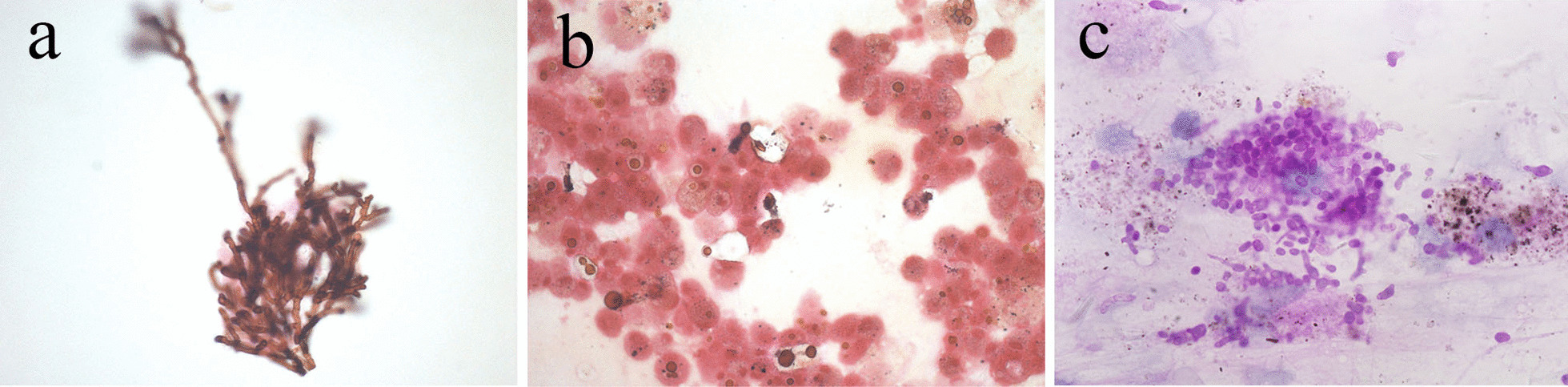
**a** Hyphae branch of Mildew repeatedly in the same direction, showing a chrysanthemum-like structure. GMSx40. **b** The cryptococcal spores are round, translucent, and thick gel-like double layered capsule, GMS stained cyst wall is dark brown. GMSx40. **c** *Talaromyces marneffei* were characterized by round or oval shape, some like a “sausage”, bearing a central dot-like structure. PASx100

The thickness of the hyphae of Mildew in the smear was the same, with transverse and acute-angled branches. They were arranged radially in the same direction and black in GMS staining. The hyphae repeatedly branched in the same direction in some areas, showing a chrysanthemum-like structure, and the cross-section was vacuous (Fig. 3a). The hyphae stained with PAS were purple-red. In the HE-staining of *Cryptococcus* BALF smears, cryptococcal spores were observed. The spores were round, translucent, thick gel-like double-layered capsule, nucleoli not obvious, and often gathered in piles; GMS-stained cyst wall was dark-brown, cell body shrunk after fixation, the cell wall was sunken, and the side was crescent-shaped (Fig. 3b). *Talaromyces marneffei* in the smear had circular or oval morphology, with some resembling "sausage" containing a central, dot-like pattern. A distinct central septum was easily observable in these yeast-like microorganisms on GMS and PAS staining (Fig. 3c).

#### In this study, the main neoplastic lesions in BALF were adenocarcinoma, squamous cell carcinoma, and small cell carcinoma, all were confirmed by tissue biopsy

**Fig. 4:**
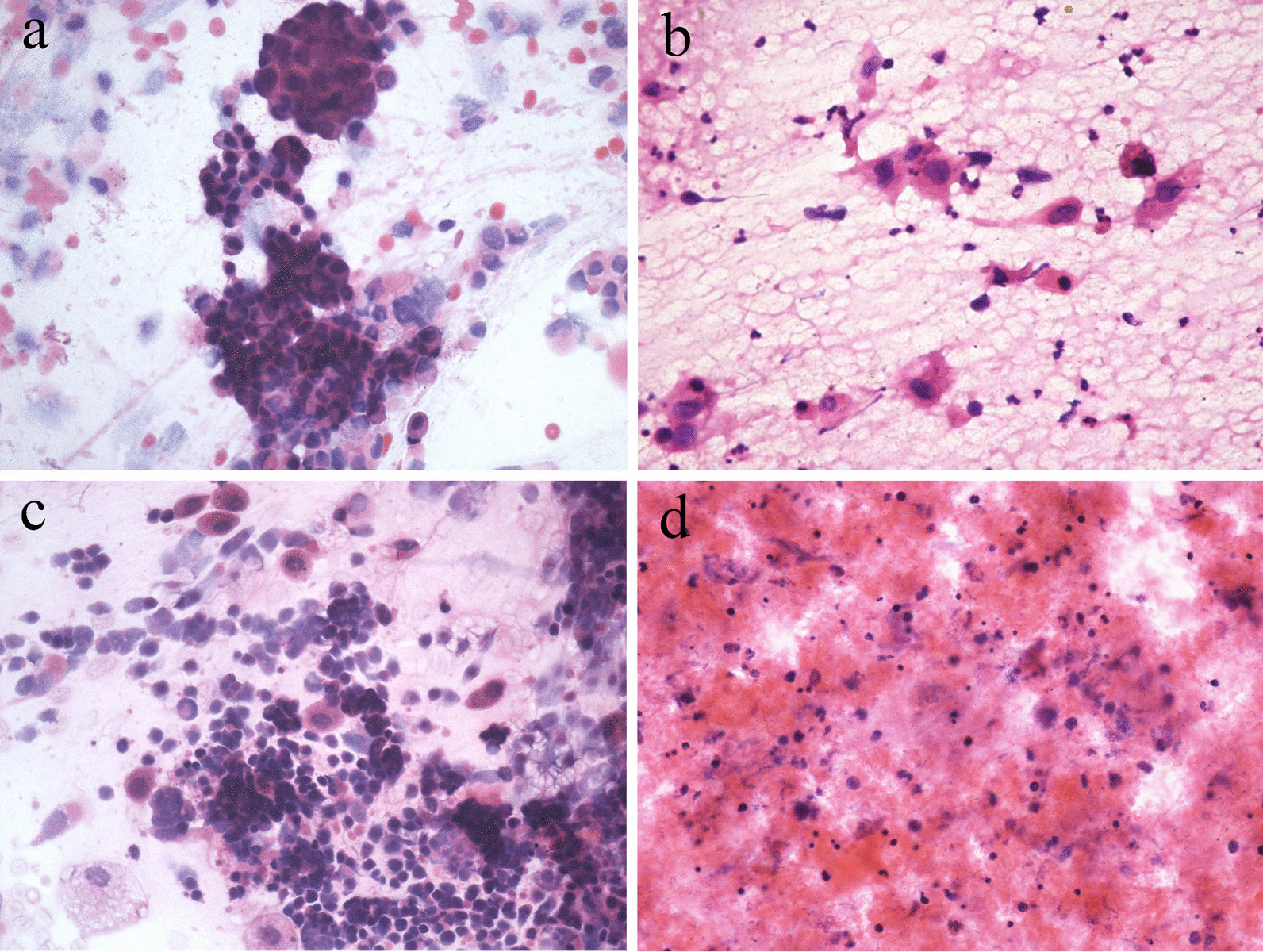
**a** Lung adenocarcinoma. scattered clusters of adenocarcinoma cells show a three-dimensional adenoid structure, with deviated nuclei, crowded arrangement, high nuclear-to-plasma ratio, coarse nuclear chromatin, and intracytoplasmic vacuoles. HEx40. **b** Lung squamous cell carcinoma. scattered squamous cells show abundant cytoplasm, enlarged and darkly stained nuclei, irregular morphology. HEx40. **c** Lung small cell cancer. Cancer cells are round or elliptical, and can be arranged into loose cell clusters. The nuclei are irregular, easily deformed and are stained in different shades, relatively fine, no nucleoli are seen. HEx40. **d** Diffuse large B-cell lymphoma. Scattered atypical lymphoid large cells in the background of necrosis. The nuclei are large and darkly stained, round or oval, with rough nuclear chromatin and nuclear debris. HEx40

In the BALF smear of lung adenocarcinoma, there were scattered clusters of adenocarcinoma cells, showing a three-dimensional adenoid structure, with deviated nuclei, crowded arrangement, high nuclear-to-plasma ratio, coarse nuclear chromatin, and intra-cytoplasmic vacuoles (Fig. 4a). In the BALF smear of lung squamous cell carcinoma, there were scattered squamous cells with abundant cytoplasm, enlarged and darkly stained nuclei, irregular morphology, and coarse and granular nuclear chromatin (Fig. 4b). In the BALF smear of small cell cancer, the cancer cells were round or elliptical and arranged into loose cell clusters. The nuclei were irregular and easily deformed. The nuclei were stained in different shades, relatively fine, and no nucleoli were observed (Fig. 4c). In addition, Diffuse Large B-cell Lymphoma (2 cases) were detected from BALF of patients with HIV infection. In the BALF smears of this lymphoma, atypical large lymphoid cells were scattered in the necrotic background. The nuclei were large and darkly stained, round or oval, with rough nuclear chromatin and debris (Fig. 4d).

## Discussion

In HIV/AIDS patients, opportunistic infections and tumors associated with HIV are major causes of death, and the lung is the most easily affected part [1]. A wide variety of pulmonary diseases can affect HIV/AIDS patients, such as opportunistic infections by numerous viral, fungal, and parasitic pathogens [2]. Recently, bronchoalveolar lavage through fiberoptic bronchoscopy has been widely used to obtain specimens. Since BALF directly comes from the lower respiratory tract, it can directly reflect the physiological and pathophysiological characteristics of the lung [3].

In this study, there were 1045 abnormalities (59.1%) in cytology smears of BALF specimens from 1768 HIV/AIDS patients, with 1635 males (92.5%) and 133 (7.5%) females, whose mean age was 38.5 years, indicating that middle-aged and young men were at high risk of HIV infection and AIDS-related lung diseases. The average age and sex ratio of HIV patients in this study were consistent with the literature reports [4]. However, there were 54 abnormalities (12.2%) in cytology smears of BALF specimens from 443 HIV-uninfected patients, consisting of 315 males and 128 females with an average age of 52.9 years. In HIV-uninfected patients, tumor lesions (40/54, 74.1%) were higher than other lesions in BALF, indicating that middle-aged and older people were at high risk of tumor lesions.

Pneumonia risk is higher in HIV infection with lower CD4 + T cell count, and several pathogens cause pneumonia [5]. Among AIDS-combined viral infections, the most frequent type is CMV infection, with the detection rate in AIDS autopsy as high as 63.1% [6]. Most infections are caused by virus activation during the late immunosuppression of AIDS patients. As a two-stranded DNA virus, the human CMV or human herpesvirus 5 (HHV-5) usually infects most individuals in their early life, which later remains lifelong in a latency. For advanced immunocompromised patients, viral reactivation is the pathogenesis of most infections. Typically, the infection occurs on the decline of CD4 + T cell count (below 50 cells/µL) among HIV-infected patients [7]. In this study, CMV pneumonia occurred in HIV-infected patients, with a detection rate of 20.9%. In addition, when there are other infectious factors, especially coexisting with PJ, CMV is often detected in the BALF [7]. In this study, PJ combined CMV infection occurred in HIV-infected people, and the detection rate was 12.5%. CMV retinitis is the most common AIDS-related ocular abnormalities, HIV infected patients presented with granular appearance of the fundus lesions with often unilateral involvement [8].

Moreover, when an HIV-infected individual shows extrapulmonary CMV disease with both lung infiltrations and other pathogens that cause pneumonia cannot be detected in the BALF, CMV pneumonia should also be suspected [9]. The methods for diagnosing CMV infection include detection of CMV antibody in serum, PCR, and virus culture from serum and BALF, and lung biopsy. CMV culture from blood and BALF has a positivity rate of 8–25% in the early stage, and the positive rate of CMV antigen in the blood is only 17% [10]. Nevertheless, the combined detection using multiple methods can significantly improve the detection rate of CMV. Culture of tracheal secretions combined with PCR of blood and tracheal secretions have a positivity rate of 35–41% [10]. We suggest that only a typical pathogenic effect ("owl's eye "cells) detected in the BALF cytology smear can be considered as the presence of CMV pneumonia. In our study, the positivity rate of CMV detected by cell morphology and PCR was 20.9%.

During the early days of the US and European AIDS epidemic, *Pneumocystis jiroveci* pneumonia (PJP) was the most common opportunistic infection among AIDS patients. About 70 to 80% of AIDS patients had PJP, and 20 to 40% died [11]. With the widespread application of anti-retroviral therapy (ART) and trimethoprim-sulfamethoxazole chemoprophylaxis (TMP-SMX), a remarkable reduction in PJP incidence was achieved among HIV-infected populations. Despite this, PJP is one major cause of opportunistic infection among these populations and is considered a serious health issue [12]. PJ belongs to the protozoan parasite, parasitic to the human alveoli. Because of the decreased CD4^+^ T cells, the immune function of AIDS patients is declined, and thus the ability to clear PJ in the alveoli is reduced, allowing it to multiply in the alveoli. The clinical symptoms and imaging features of PJP are not obvious, and the diagnosis is difficult. The definitive diagnosis of Pneumocystis often relies on a histopathology or cytopathology sputum or BALF demonstration since *in-vitro* culturing of the organism is impossible, and the diagnosis is confirmed under a microscope after special staining. A few techniques are available for staining the organism. The pathological changes of PJP are mainly diffuse exudation of eosinophilic serous fluid and foam-like changes in the alveoli, with several cysts. The BALF smear shows a focally distributed pink foamy or honeycomb-like substance containing piles of PJ Cyst. GMS staining is currently recognized as a staining method to determine PJ [13]. In addition, Immunocytochemical staining of BALF smears with specific antibodies against PJ can also be used, but the positive rate is not high. With the progress in PJP genome research, primers can be designed for different subtypes, and the PCR method for detecting different PJP subtypes can be successfully achieved. Molecular detection of Pneumocystis in BALF has become an essential diagnostic tool. The clinical PJP diagnosis has improved, owing to highly specific and sensitive PCR instruments enabling precise early diagnosis of Pneumocystis infections, which should shorten the duration from symptom onset to therapy, a prognostically significant period [14].

HIV combined tuberculosis infection is in the top ten causes of death globally. The high mortality is significantly attributed to the interaction of these two pathogens. Tuberculosis infection is the main opportunistic infection and cause of death of HIV-infected individuals [15]. The possibility and mortality rate of HIV-infected people who develop the active disease after being infected with tuberculosis is significantly increased. Pulmonary tuberculosis is the most common form of tuberculosis in HIV-infected persons. Some studies showed that HIV-infected persons are more likely to have "non-classical" or atypical chest radiographs compared with HIV-negative persons. HIV-positive individuals with normal CD4^+^ T cell counts manifest traditional symptoms of pulmonary TB, such as pleural effusions and lymph node lesion, while few symptoms restricted to the pulmonary apices also occur. In advanced stage AIDS, mycobacteraemia and disseminated extrapulmonary lesion are often caused by *M. tuberculosis* [16]. In this study, the detection rate of mycobacteria was only 3.67%, which was lower than the incidence of extrapulmonary lymph node tuberculosis in our previous study [17], partly due to the lack of tuberculosis cavity lesions. In addition, because sub-clinical tuberculosis is inactive, the amount of tuberculosis bacteria obtained by the lavage fluid is less, which further increases the difficulty of detection [18]. As the disease progresses, most HIV patients with subclinical tuberculosis symptoms will eventually have typical symptoms of tuberculosis. When HIV-infected persons with subclinical tuberculosis start anti-retroviral therapy, they may also have typical symptoms of tuberculosis because their immune system is restored, which is called immune reconstitution inflammatory syndrome (IRIS) [19]. At this time, the detection rate of mycobacteria is increased in BALF.

Non-tuberculous mycobacteria (NTM), another member of the *Mycobacterium* genus aside from *Mycobacterium leprae* and Mycobacterium tuberculosis complex, are widespread in both natural and anthropogenic environments, primarily in water, soil, and biofilms. NTM can induce various diseases in humans, especially affecting the lungs [20]. Overall, in industrialized countries, the incidence of MAC infection in general and HIV populations is increasing while the incidence of tuberculosis continues to decrease [21]. The most reported NTM infection in HIV patients was Mycobacterium avium complex and *M. kansasii*[20]. In this study, MAC, *M. kansasii,* and *M. szulgai* were detected in BALF of HIV-infected patients. PCR reverse dot blot hybridization was used to identify 22 species of *Mycobacterium*.

Many resource-abundant regions showed a reduced incidence of systemic fungal infections among HIV patients after introducing ART and earlier HIV diagnosis and therapy. However, such incidence is yet to decline in many resource-scarce settings, where the diagnosis is persistently late, and there are challenges with HIV retention care [22]. Three main fungi groups cause lung infections in HIV patients: *Aspergillus*, *Cryptococcus neoformans*, and *Biphasic fungi* [7]; all found in our study. *Aspergillus* is ubiquitous, especially *Aspergillus fumigatus*, which most frequently infect the HIV population [23]. Neutropenia, glucocorticoids application, CD4^+^ T cell count less than 50 cells/µL are significant risk factors for invasive aspergillosis [23]. Cell smears or tissue sections with mold hyphae are the basis for diagnosis. *Aspergillus* hyphae are light blue translucent, with consistent thickness, septa, and acute-angled branches arranged radially in the same direction and are highly invasive. Cryptococcal infection is mainly caused by spore aspiration or yeast cells, and a pulmonary lymph node syndrome is formed during the early infectious period; the form is like tuberculosis [7]. Cryptococcal infections in HIV patients are caused by latent infections or activation of primary infections. Most cryptococcal infections occur among individuals having less than 200 CD4^+^ T cells/µL, and a CD4^+^ T cell count of ≤ 100 cells/µL is recommended as the routine criterion by the World Health Organization for screening cryptococcal infection of HIV/AIDS patients [24]. Cryptococcal infection is usually manifested as disseminated infection with meningitis in HIV-infected patients. In immunological terms, cryptococcal meningitis is expected to be more prevalent at lower instead of higher levels of CD4^+^ T cells, given the worse immunosuppression and weaker infection controllability of individuals [25]. Lumbar puncture is a must for all HIV/AIDS patients contracting cryptococcal pneumonia to exclude the central nervous system involvement. Biphasic fungi mainly include *Histoplasma* and *Penicillium marneffei*, which produce infectious soft wart-like skin lesions, hepatosplenomegaly, and lymphadenectasis. The diagnosis is confirmed by skin biopsy and lymph node puncture smear. Our previous study found *Histoplasma* and *Penicillium marneffei* infection in lymph node puncture smears [17].

The clinical types and flora distribution of lung infections in HIV/AIDS patients differ from those with normal immune functions. Multiple pathogens are often present simultaneously, and the infection spectrum is complex and changeable [26]. The HIV/AIDS patients were clinically present with non-specific lung infections, whose imaging demonstrations were diversified. The diagnosis of any specific pathogen cannot be confirmed and may overlap with non-infectious lung diseases. Therefore, bronchoscopy and cytology smears are required to confirm the diagnosis.

In addition to AIDS-related opportunistic infections, another notable feature of the AIDS population is the increase in malignant tumors. Since people began to recognize AIDS in the early 1980s, they noticed that certain tumors are closely related to AIDS. Among them, the most common AIDS-associated malignancy is Kaposi's sarcoma (KS), but its incidence dropped significantly [11] due to anti-retroviral therapy. One-third of KS clinically has lung involvement, but KS was not found in this group, and we previously found only 4 cases in 453 lymph node lesions [17]. For AIDS patients without mucocutaneous involvement, the difficulty of diagnosing KS increases due to the usually co-prevalent opportunistic infections or other tumors. Despite being the most sensitive procedure for pulmonary KS evaluation, bronchoscopy can hardly accurately diagnose when the typical endobronchial lesions are absent [27]. The second most prevalent AIDS-associated malignancy is Non-Hodgkin's lymphoma (NHL). Studies have shown that 30% of AIDS-related NHL patients have various pulmonary complications [11]. This group found 2 cases of non-Hodgkin diffuse large B-cell lymphoma. Among the HIV-positive patients, NHL is deemed either a secondary body/extension of the extrapulmonary origin or primary pulmonary NHL, a distinctive form. The occurrence of primary pulmonary lymphoma is scarce in immunocompromised and immunocompetent individuals, and its morbidity is unknown, especially among HIV-infected individuals [28].

Lung cancer has become the most common non-AIDS-related malignant tumor in HIV-infected individuals. Some epidemiological studies have shown that the incidence and mortality of lung cancer among HIV-infected patients tend to increase, which may be due to the longer survival time of patients with anti-retroviral therapy, leading to an increase in the incidence of lung cancer [29]. This group showed 11 cases of lung cancer, including adenocarcinoma (4 cases), squamous cell carcinoma (2 cases), and small cell carcinoma (3 cases). Pulmonary cancer occurs among HIV-positive patients at 25–30 years of age on average, earlier than the general population, and besides, there were few smokers. Although anti-retroviral therapy can increase the CD4^+^ cell count and postpone the disease progression, the outcome is still not good [30].

## Conclusions

A significant difference in the spectrum of lung diseases between HIV-infected and non-infected people, CMV, PJ, Fungal, and Mycobacterial infections are the main findings in HIV/AIDS-related lung disease. Double and Triple infections were found only in HIV-infected patients. Thus, BALF cytopathology provides pathogen morphological evidence and has significant clinical value.

## Data Availability

The datasets used and/or analyzed during the current study available from the corresponding author on reasonable request.

## Abbreviations

HIV: Human immunodeficiency virus
AIDS: Acquired immunodeficiency syndrome
BALF: Bronchoalveolar lavage fluid
IHC: Immunohistochemical
HE: Haematoxylin and eosin
PAS: Periodic acid schiff
GMS: Gomori’s methenamine silver
TB: Tuberculosis
MAC: Mycobacterium avium complex
NTM: Nontuberculous mycobacterium
PJ: Pneumocystis jiroveci
PJP: Pneumocystis jiroveci pneumonia
